# Adverse Drug Reactions during COVID-19 Treatment: A Comprehensive Analysis Focused on Hospitalized Patients, with the Use of a Survey in Cuba in 2020

**DOI:** 10.1155/2023/1995642

**Published:** 2023-02-01

**Authors:** Lizette Gil-del-Valle, Rosario Gravier-Hernández, Waldemar Baldoquin-Rodríguez, Beatriz Sierra-Vázquez, Ana Beatriz Perez-Díaz, Pablo Sariol-Resik, Tatiana Prieto-Dominguez, Mario Manuel Delgado-Guerra, Joniel Arnoldo Sánchez- Márquez, Olga Elena López-Fernández, Faustina Fonseca-Betancourt, Liana Valdés-Lanza, Odalys Orraca-Castillo, Xaveer Van Ostade, Wim Vanden Berghe, Veerle Vanlerberghe, M. Guadalupe Guzmán-Tirado

**Affiliations:** ^1^Institute “Pedro Kourí” (IPK), Havana, Cuba; ^2^Hospital Ernesto Guevara, University of Informatics Science, Havana, Cuba; ^3^Universidad de Ciencias Médicas de Pinar del Río, Pinar del Río, Cuba; ^4^University of Antwerp, Department of Biomedical Sciences (BMW), Faculty of Pharmaceutical, Biomedical and Veterinary Sciences (FBD), Antwerp, Belgium; ^5^Institute of Tropical Medicine, Antwerp, Belgium

## Abstract

**Aims:**

To evaluate the prevalence and type of adverse drug reactions (ADRs), together with associated risk factors, among Cuban COVID-19 patients treated with chloroquine (CQ), lopinavir/ritonavir (LPV/r), or interferon *α*2b (IFN *α*2b), according to the Cuban protocol.

**Materials and Methods:**

A prospective descriptive analysis of ADRs was performed on 200 COVID-19 patients who were admitted consecutively to three hospitals in Havana and Pinar del Río from April to July 2020. Information on demographics, ADRs, outcomes, behavioral, and health-related factors was collected using a validated questionnaire and clinical records. Each potential ADR case was assessed for causality based on the WHO-UMC algorithm, concomitant drug influences, and the presence of any drug-drug interactions (DDI).

**Results:**

The total frequency of ADRs was 55%, with predominantly gastrointestinal disorders and general symptoms (23% vs 20%). 95.1% of ADRs occurred within 10 days after treatment and 42 potential DDI in 55.5% of patients (61/110) were observed. The prevalence of ADRs was: 44%, 30.4%, and 26.4% for IFN *α*2b, LPV/r, and CQ, respectively. Sex (odds ratio (OR): 0.40 (95% confidence interval (CI): 0.211–0.742), age (OR: 2.36 (95% CI: 1.02–5.44)), and underlying diseases (OR: 0.12 (95% CI: 0.06–0.23)) were independently associated factors for ADRs (*P* < 0.05).

**Conclusions:**

The frequency of ADRs and potential DDI was high compared to their use during nonpandemic times (e.g., for malaria, HIV, or inflammatory diseases). The safety profile of these drugs when used for COVID-19 treatment showed similar characteristics. Comorbidities, age >37 years old, and female sex were associated with ADRs.

## 1. Introduction

SARS-CoV-2, discovered in Wuhan, China, is a highly contagious pathogen that spread quickly throughout the world, and which caused a pandemic of global concern. According to the World Health Organization, 223 countries have been affected, with nearly 250 million people infected worldwide. There is a lack of a specific treatment for SARS-CoV-2-induced severe acute respiratory syndrome, but effective vaccines became available by late 2020 [1–3].

People infected with SARS-CoV-2 can be asymptomatic or can present symptoms with varying severity, which is possibly associated with the patients' age and underlying comorbidities. The most commonly reported symptoms are as follows: fever, dry cough, headache, myalgia, fatigue, and diarrhea. SARS-CoV-2 causes marked inflammatory and immune responses that may activate a “cytokine storm,” which results in apoptosis of epithelial and endothelial cells and, subsequently, vascular leakage and abnormal T cell and macrophage responses ensue [4, 5].

Many host cellular factors have been identified that are involved in the viral infection and in the various steps of the lifecycle or viral products that interfere with the homeostasis of cell metabolism. These factors cause significant cellular stress to the host cell, which induces cell and tissue damage [6].

Since the beginning of the pandemic, there have been several potential drug candidates for COVID-19 treatment, such as lopinavir/ritonavir, interferon alpha 2b, umifenovir, chloroquine, remdesivir, favipiravir, anti-inflammatory drugs (such as corticosteroids and other molecules), and traditional medicines. These were considered to be drug repurposing or off-label, compassionate use drugs because there was a lack of clinical trial data supporting the use of these drugs in COVID-19 [7, 8]. Hence, these drug treatment schemes and posologies must be carefully investigated to maximize their benefits with minimal toxicity or adverse drug reactions. Previous reports have shown that the treatment of COVID-19 patients with preexistent, noncommunicable diseases was complex [9] and that they had an increased risk for drug-related adverse reactions [10, 11].

In early 2020, in Cuba, the option of drug repurposing and off-label, compassionate use was discussed and approved by an ad hoc commission of the Cuban Research and Science Ministry of Health. A consensus protocol of case management and treatment was approved in March 2020, which included compassionate use of lopinavir/ritonavir, interferon *α,* and chloroquine for all hospitalized patients that tested positive on a SARS-CoV-2 RT-PCR test, whether they were asymptomatic or not [12].

The recommended drugs, such as HIV protease inhibitors, interferon, and chloroquine have previously shown complex DDI [13, 14]. Drug safety must never be ignored, especially when there is still doubt about the drug's efficacy against the pathogen, as was the case for COVID-19. The drug's benefits must always outweigh its risks [15]. ADRs are described for the three recommended drugs, and they range from mild to life-threatening and short- to long-term effects, according to previous use worldwide [10, 11, 16]. Hence, evaluation of potential ADRs for the drugs used for the Cuban COVID-19 protocol was performed [12]. Little is known about the incidence of ADRs to these drugs when used in COVID-19 patients. In the literature, ADRs reaching 20–40% are reported when used for HIV, malaria, and inflammatory diseases [10, 11, 16] but 40–70% of ADRs are reported in COVID-19 patients [9, 13, 17]. In low- and middle-income countries, the main priority is securing access to drugs for treatment, which leaves limited resources for pharmacovigilance [18, 19]. Thus, it is clear that drug safety data should be collected and analyzed to obtain the necessary information for decision-making, to manage potential risks associated with certain drugs or their combinations.

The “Institute Pedro Kouri” hospital in Havana, Cuba, is taking part in the Sentinel Surveillance on Pharmacovigilance. A specific system was set up, where patient notification reports are routinely collected and analyzed for potential ADRs in infectious disease treatments [20]. This sentinel surveillance system aims to solve the problems of underreporting, undue delays, and miscommunication that were observed in the previous passive surveillance system on ADRs.

Better reporting systems and the rapid analysis of information will have a positive effect on rational drug use in hospitals [21]. In this study, we characterized the ADRs during the first wave of COVID-19 in Cuba and analyzed possible drug-drug interactions. Our results obtained on drug safety will provide a solid basis for decision-making by the Cuban Ministry of Health about drug risk management in COVID-19 treatment. Moreover, our data are important for enhancing current treatment regimens by reconfiguring the recommended therapeutic combinations and to provide potential information for personalized treatments.

## 2. Materials and Methods

This study was done in accordance with the principles of the Declaration of Helsinki and the Good Clinical Practice Guidelines of the International Conference on Harmonization. The study protocol was approved by the Scientific and Ethics Committee (by fast-track) of IPK, University of Informatics Science Hospital (UISH), and Pinar del Río Medical Science University Hospital (PRMUH), Cuba, on May 17, 2020. This study was also approved by and registered at the Cuban Ministry of Health (code number 2105028) in accordance with the regulatory Cuban agency (CECMED).

### 2.1. Setting

The first cases were reported in Cuba in March 2020, and in accordance with the COVID-19 treatment protocols that were approved in April 2020 by the Cuban Ministry of Health Commission, all RT-PCR positive patients were admitted to different hospitals. All these hospitals were prepared with the necessary COVID-19 biosafety precautions. All patients were hospitalized and treated during acute COVID-19 with the three drugs: Kaletra (lopinavir/ritonavir), chloroquine, and interferon *α*2b, regardless of their clinical diagnosis (i.e., asymptomatic or symptomatic for COVID-19). COVID-19 cases were tracked by contacts with index cases, and all were isolated and tested with rapid tests or/and PCR. If patients tested SARS-CoV-2 positive, they were hospitalized and could be included in this study, dependent upon their time of presentation and admission to the hospital. All COVID-19 patients were hospitalized for the full duration of their treatment (30 days).

Inclusion criteria: all adult patients (>18 years old) with a SARS-CoV-2 positive RT-PCR test were included, with no sex restrictions. An exclusion criterion was participation in another clinical trial within the last three months.

All patients with SARS-CoV-2 infection confirmed by RT-PCR and hospitalized in IPK, UISH, or PRMUH were consecutively included in this study from April to July 2020.

There was no sample size estimated, as all patients in the study locations who gave consent were included in the study.

### 2.2. Questionnaire

To evaluate the factors associated with ADRs, a 105-point paper-based questionnaire was used, which explored factors related to 11 domains, such as general health, demographics, and risk behaviors.

Aspect 1-patient demographic characteristics (age, sex, and ethnic group); Aspect 2-patient clinical status; Aspect 3-suspected drugs and concomitant medications (for each one, administration route, therapy duration, dosage, and therapeutic indication were recorded); Aspect 4-ADR description; Aspect 5-ADR outcome (improvement, complete resolution, unchanged or worsened event, resolution with sequelae, and death).

This survey is a modified version of the questionnaire on “Lifestyle Related Practices and Beliefs,” designed, validated, and applied by Arrivillaga et al., to describe the health-related beliefs of a community of young university students in Cali, Colombia. It also assessed their attitudes toward risk and protective behaviors [22]. We obtained permission from Arrivillaga et al. to make a modified version of their questionnaire.

Four members of our research team were trained as interviewers to ensure uniformity in data collection. When conducting the survey, they introduced themselves to the participants and explained by phone the purpose of the survey. Data related to aspects 1 and 2 were obtained from health records. Data for aspects 3, 4, and 5 were collected by phone. The pharmacologists and physicians who reviewed the ADRs were specifically involved in this study for this purpose. A “suspected drug” is defined as a drug which is potentially associated with the observed ADRs, while a “concomitant medication” is a drug that the patient is exposed to at the time of ADR occurrence. A concomitant medication may not necessarily be associated with the ADRs. Moreover, each case was evaluated with the aim of identifying the presence of DDI, which may have contributed to ADRs. DDI were identified using the open access Drug Interaction Checker. For each case, the physicians consulted the source documents that were archived in the hospital medical record system.

A detailed written explanation of the questionnaire process, the researchers' names and institutions, and possible benefits for the patients and others were clearly explained to the participants, and additional information was provided upon request. Informed participants were asked to sign a written informed consent. All patients were codified and anonymized to protect the confidentiality of individual participants.

Two hundred patients with SARS-CoV-2 infection, confirmed by RT-PCR, during April to July 2020, that were hospitalized (72 in IPK, 38 in UISH, and 100 in PRMUH), aged 19–85 years old at the time of inclusion, were interviewed by phone. The questionnaire was performed while the patients were in the hospital, just before their discharge from the hospital. ADRs are defined by the WHO as noxious and unintended responses to drugs at standard doses. The ADR reports were reviewed by pharmacologists and physicians that were specialized in pharmacovigilance. All the data collected for ADRs and their further characterizations were sent to the National Pharmacovigilance Unit.

### 2.3. Treatment Regimen

All patients received the three drugs daily according to the following regimen: Kaletra (200 mg lopinavir and50 mg litonavir) 2 capsules per 12 hours for 30 days, chloroquine (250 mg = 150 mg base) 1 tablet per 12 hours for 10 days and 3 million units of interferon *α*2b, by intramuscular injection, 3 times a week for 4 weeks.

### 2.4. Data Collection

Other characteristics of the COVID-19 patients (time of admission, length of stay, sex, age, disease diagnoses, etc.), history of drug allergies, the antiviral protocol, and the number of medications used during hospitalization were extracted manually from the study questionnaires.

### 2.5. Case Assessments

Causality assessments were performed for all suspected ADRs using the World Health Organization-Uppsala Monitoring Centre (WHO-UMC) system. The WHO-UMC system is a universally accepted method for causality assessment [23].

The seriousness of the identified suspected ADRs was determined according to the definition of the ICH E2A guideline (ICH E2A Clinical Safety Data Management: Definitions and Standards for Expedited Reporting), which includes and combines the severity classification that is evaluated with Hartwig's Severity Assessment Scale as mild (levels 1 and 2), moderate (levels 3 and 4), and severe (levels 5–7) [24]. The Hartwig's Severity Assessment Scale contains the following levels: level 1: an ADR occurred but required no change in treatment with the suspected drug; level 2: the ADR required that treatment with the suspected drug be held, discontinued, or otherwise changed. No antidote or other treatment requirement was required. No increase in length of stay (LOS); level 3: the ADR required that treatment with the suspected drug be held, discontinued, or otherwise changed. AND/OR an antidote or other treatment was required. No increase in LOS; level 4: any level 3 ADR which increases length of stay by at least 1 day. OR the ADR was the reason for the admission; level 5: any level 4 ADR which requires intensive medical care; level 6: the adverse reaction caused permanent harm to the patient; level 7: the adverse reaction either directly or indirectly led to the death of the patient. The relationship between the reported ADRs and drugs was categorized as certain, probable, possible, unlikely, conditional/unclassified, or unassessable/unclassifiable. Only cases categorized as certain, probable, and possible were included in our study.

### 2.6. Potential Drug-Drug Interactions

For the determination of potential DDI among chloroquine, Kaletra, interferon *α*2b, and other concomitant drugs, the Medscape and Liverpool Interaction Checker (https://reference.medscape.com/drug-interactionchecker) webpages were uniformly consulted, as recommended in the national and international treatment guidelines [25, 26]. Potential DDI were those classified according to risk level: *X* (contraindicated association), *D* (consider modification of therapy), and *C* (monitoring therapy). Subsequently, the DDI frequency was analyzed and each interaction was described according to its mechanism of action, the level of risk, if it affected LPV/r, the other drug involved in the interaction, or both, if it could compromise its efficacy or cause toxicity. and the recommendation in each case.

### 2.7. Data Processing and Statistical Analyses

Data were stored digitally using an entry program developed with the WPS software package. All data were processed using Microsoft Access. Both descriptive and analytical analyses were carried out on the data using Microsoft Excel and Version 22, SPSS Inc., Chicago, IL, USA.

Bivariate analysis was carried out to evaluate the associations of potential risk factors with the presence of ADRs. Results were presented as percentages and frequencies. To test statistical significance, 95% confidence intervals and/or *P*-value were used. A *P*-value <0.05 was considered to be statistically significant.

## 3. Results

### 3.1. Patient Characteristics

Two hundred (200) patients were included in this study (Table 1). From these, 94 (47%) were female and 106 (53%) were male. The mean age was 46 years old with (95% CI 20–76). Underlying diseases such as hypertension, cardiovascular disease, diabetes, asthma, cerebrovascular disease, cancer, chronic kidney disease, chronic liver disease, obesity, epilepsy, arthritis, and others were identified in 70.5% of them. 72.5% of all patients were Caucasian.

Of the 200 patients, 65 showed asymptomatic progression of COVID-19, and 135 presented low or moderate symptoms. A total of 232 ADR manifestations were identified in 110 patients. The overall cumulative incidence of ADRs was 55%.

The median age of the patient group without ADRs and with ADRs was 56 (95% CI 34–75) and 41 (95% CI 20–64) years, respectively. The number of supportive drugs used in the hospital (OR 2.36 (*P* < 0.05)) was significantly higher in the ADRs group (Table 1). There were no significant differences in the proportions of skin color, toxic habits (alcoholism and smoking), and COVID-19 progression between the two groups. The results of the bivariate analysis are also shown in Table 1. The independent risk factors for the occurrence of ADRs in Cuban COVID-19 patients were age higher than 37 years old (OR:2.36 (95% CI:1.02–5.44), *P* = 0.043), female sex (OR: 0.40 (95%CI: 0.21–0.74), *P* = 0.004), and underlying diseases (OR:2.07 (95%CI: 1.02–4.23), *P* = 0.0001).

### 3.2. Characteristics of ADRs

**Figure 1:**
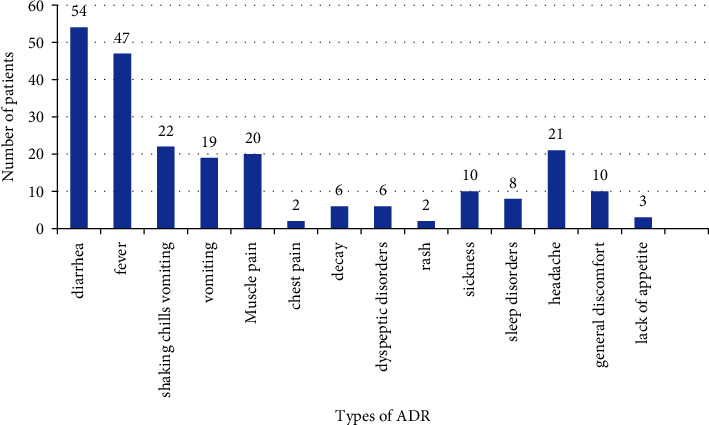
Type of ADRs observed in Cuban COVID-19 patients hospitalized in HEG-UCI, IPK, and Pinar del Río. Source: completed surveys that were accessed in a digital database and printed surveys. ADR, adverse drug reaction. COVID-19, Coronavirus disease 2019. HEG-UCI: Hospital Ernesto Guevara at University of Informatics' Science (HEG-UCI acronyms in Spanish) IPK-Institute Pedro Kourí.

Drug-related adverse reactions, as categorized by manifestations, are shown in Figure 1. Gastrointestinal (GI) disorders (diarrhea, vomiting, and nausea) were the most frequent ADRs with an occurrence of 23.0%, followed by general symptoms (fever, pain, decay, and general discomfort). For the onset of the ADRs, 9.57% (rash, diarrhea, and vomiting) occurred within the first day of treatment, 79.8% occurred within 7 days of treatment, and 95.1% occurred within 10 days of treatment.

Suspected drugs with ADRs were associated with interferon *α* and lopinavir/ritonavir with 44% and 30.4%, respectively. CQ caused the lowest ADR incidence with (29/110) 26.4%, of which only 7.3% were found to be serious (Table 2). The causal relationship assessed by using the WHO-UMC system in the suspected ADR cases was found to be probable and possible (76% vs. 24%). The prognoses of ADRs in these patients were favorable, with 75% cured and 25% improved.

Other drugs (in total, 63) used by patients for treatment of comorbidities are listed in Table 3, as well as the frequencies of DDI by drug, together with the descriptions of each interaction according to the following lists: mechanism of action, risk level, effect, and recommendations. Potential DDI with prescribed drugs for COVID-19 treatment was found in 61 patients using, in total, 29 drugs, which represent 46.0% (29/63). 55.5% of patients (61/110) who presented ADRs and comorbidities suffered from DDI. A risk level *X* was identified in only 1 patient, the risk level *D* was identified in 30 of 61 cases (49.2%), and the risk level C was observed in 30 of 61 cases (49.2%) (Table 3). The drug with the highest interaction frequency was lopinavir-ritonavir (*n* = 59), followed by CQ (*n* = 23). With respect to interferon *α*2b, no interactions were identified, in concordance with the low or nonreports previously described for the studied drugs (41, 42). An exception was hydrochlorothiazide, which potentially interacts with interferon *α*2b, CQ, and ritonavir/lopinavir. Only one type of DDI was identified involving CQ, while 17 DDI were related to lopinavir/ritonavir. Potential interactions related to both CQ and LPV were identified for 10 supportively prescribed drugs (45) (Table 3).

## 4. Discussion

The COVID-19 pandemic has overwhelmed healthcare systems worldwide. A more detailed stratification of the adverse-related events of prescribed drugs to treat COVID-19 is required [8]. In this study in Cuba, we used for the first time a comprehensive survey to identify potential drug safety issues for off-label drugs that were used to treat COVID-19. We aimed to obtain evidence for a specific combined drug approach that could be used for the prevention and treatment of COVID-19.

Our study actively recorded ADR symptoms in hospitalized patients in hospitals in Cuba under continuous monitoring, and data were directly reported to the National Pharmacovigilance Centre, to avoid any potential under-reporting or miscommunication. As all patients with a positive RT-PCR test were hospitalized in Cuba, this provided an ideal opportunity to obtain data on the real incidence of ADRs in COVID-19 patients [18]. We ensured that there was no bias in the inclusion of COVID-19 patients in this study, so our study results may also be applicable in other contexts. Some of the main reasons for nonreporting in other settings included: a lack of time, no access to documentation, insufficiently trained personnel, the personnel being busy with other tasks, failure to recognize an ADR, patient confidentiality concerns, biosafety resolution regarding clinical history use (quarantine), and fear of blame. Our study showed that ADRs in COVID-19 patients were mainly characterized by GI reactions and general symptoms such as fever and general discomfort, with an incidence of 23.0% and 20%, respectively. ADRs that occurred within 10 days represented 95.1%. As ritonavir/lopinavir and chloroquine were orally administered, the GI manifestations associated with these drugs typically occurred within 7 days. Fever was presumably related to injections of interferon *α*2b, but this was not exclusive, and it typically appeared within 24 to 48 hours.

Various studies on drug safety monitoring in COVID-19 patients have been published [9, 13, 15, 17, 27–31]. One study in China reported diarrhea that was associated with lopinavir/ritonavir use in COVID-19 [17] and showed that after using lopinavir/ritonavir in 33 patients with COVID-19 in the Nanchong area, 15 patients had diarrhea, rash, and other adverse reactions, with an incidence of 42.9%. In our study, the incidence of ADRs by lopinavir/ritonavir was 30.4% (34/110), which was lower than studies from China [9, 17] but higher than the Melo study from Brazil [30] and the Maza Larrea study from Mexico [31]. The proportion of ADRs caused by interferon *α*2b in our study (44%) was the highest, which was similar to other COVID-19 studies [9, 30].

Results obtained from other studies differ slightly from ours. This may be due to differences in population, context, and sex-age composition [32], or ancestry genotypic polymorphisms as have been observed in other viral diseases in Cuba [33]. The safety monitoring data obtained in previous studies for lopinavir mainly focused on HIV patients and healthy people [13]. A randomized trial to evaluate the efficacy and safety of lopinavir/ritonavir in the treatment of HIV-1 infection reported that the incidence of diarrhea was high (26/40, 65%), resulting in 16% of moderate/severe adverse events [13]. Moreover, recent COVID-19 studies presented GI alterations as the main ADR [1, 13, 15].

In our study the incidence of adverse reactions by chloroquine was 29/110, and mainly manifested as GI reactions, general discomfort, and headache. No decreases in vision or cardiotoxicity were observed, which was in contrast to other studies [9, 13, 32], as this is induced by long-term use of chloroquine, which was not the case in our study as it was generally administered for less than 30 days [24].

After symptomatic supportive treatment, 75% of ADRs were cured and 25% improved. Only 28% (38/110) of patients interrupted their treatment (21/110 using interferon *α*2b, 12/110 using Kaletra, and 5/110 using chloroquine).

In clinical trials with lopinavir/ritonavir recipients, hypertriglyceridemia and hypercholesterolemia were the most frequently observed alterations and were mainly seen in the first month of treatment [27], which represents a potential reason for discontinuation of therapy [13]. This supports the need to closely monitor potential changes in blood lipids in patients undergoing treatment.

In our study, ten serious ADRs were observed in patients who each suffered from 3 or 4 underlying diseases. All these patients used polypharmacy with 5 potential DDI related to both chloroquine and ritonavir-lopinavir, had toxic habits (alcoholism and smoking), and showed ages higher than 37 years. These patients discontinued the drug therapy, after which their symptoms resolved in 1 to 3 days.

As ADRs are the most common reason for poor adherence to treatment, identification of risk factors for the occurrence of ADRs is essential for optimizing the initial choice of drug regimen. Our study showed that the independent risk factors for the occurrence of ADRs in COVID-19 patients included age (OR: 2.36 (95% CI: 1.02–5.44), *P* = 0.0059), sex (OR: 0.40 (95% CI: 0.21–0.74), *P* = 0.0038), and underlying diseases (OR: 0.21 (95% CI: 0.11–0.40), *P* = 0.0001).

The majority of ADRs observed in our study are in correspondence with previously reported adverse reactions or the recognized safety profiles for these drugs [10, 11, 24]. Only 2 ADRs were identified as rare and 3 as occasional in our study.

In accordance with our study, a significant association between age, sex, and comorbidities and the occurrence of ADRs was also observed in studies by Kojima et al. [34], Bandariz et al. [35], and Jing et al. [9]. Alterations in age are typically concomitant with underlying changes in the human body, such as a reduction in renal clearance, liver size, lean body mass, and other factors which may impact biological processes and, in consequence, alter drug distribution, metabolism, and pharmacodynamic responses [36].

In our study, an association of ADRs and sex was found, which was in concurrence with the results obtained by Jing et al. for lopinavir/ritonavir and Zekarias et al. for CQ and lopinavir/ritonavir [9, 28]. This could be explained by differences in fat distribution, greater plasma volume, organ perfusion, and hormone-related regulation of drug metabolizing enzymes, as has been reported in other studies [37].

Preexisting or underlying diseases, or comorbidities, have also been identified as associating factors. Comorbidities produce pathophysiological changes that could have an effect on drug pharmacodynamic and pharmacokinetic properties, and hence patients could be given unsuitable drug prescriptions [36]. Polypharmacy has been reported to be a strong risk factor for ADRs in several studies in relation to possible DDI [1, 18]. Potential DDI was identified in 63 patients for 29 drugs, mainly previously prescribed for COVID-19 infection, and could be a factor contributing to the high ADRs observed.

The DDI identified in our study are potentially related to the modifications in drug concentrations, and this could affect their antiviral activity or influence on comorbidities. The DDI could also cause an increase in toxicities. Indeed, for 28 of the 29 drugs used, it is strongly recommended to monitor or avoid their use due to increased toxicities. For only one drug, concomitant administration with CQ, IFN, and LPV/r was contraindicated.

Different studies have reported that ADR incidence in hospitalized patients varies from 8 to 35%, which was mainly based on the physicians' spontaneous reports [14, 18]. The high incidence observed in our study (55%) could also be related to the nonexplored interactions among drugs-host-viruses in the new pandemic scenario that required an in-depth study of the ADR-DDI in COVID-19 patients [14, 32, 35]. Some researchers have suggested that the application of a survey could be a useful method for collecting patient reported ADRs [21]. Reporting by the patients generally implies the use of the Internet or phone. For those patients who are not comfortable using Internet tools or where computers are not available in the hospitals involved in the study, another option should be made available, such as reporting by phone using a survey adapted to that case [21]. The last variant was the one used in our study.

The vast majority of COVID-19 patients in previous studies were hospitalized because of fever [9, 15, 17]. Hence, in this study, this bias was not present, as most of the hospitalized individuals were nonsymptomatic (contact tracing individuals). In univariate analysis from previous studies, the length of the hospital stay was reported to be significantly associated with the occurrence of ADRs [27, 31]. No association was found in our study in relation to the duration of hospital stays, which was likely because hospitalization and duration of hospitalization were not dependent upon symptomatology or severity of infection.

Toxic habits such as alcoholism and smoking, which have a high incidence in the Cuban population, could also impact ADRs, but we found no association between ADRs and these toxic habits.

The interpretation of our results is limited to the context from which participants were recruited. We mainly studied COVID-19 patients that were hospitalized in occidental provinces, which may not be representative for patients hospitalized across different regions in Cuba. In addition, a partial evaluation of possible ADRs does not completely exclude the influence of COVID-19. In our study, both physicians and researchers made an effort to carefully make a distinction between drug effects and COVID-19 infection symptoms. Altered medical parameters and symptoms observed in patients, related to preexistent diseases different from COVID 19, cannot be included as ADRs. Furthermore, we only observed ADRs during hospitalization, after the drugs had been administered [30]. Also, late-stage retinopathy caused by chloroquine may occur many years after discontinuation of the treatment. Further studies with longer follow-up periods are required to assess the longer-term implications of ADRs. For DDI, some discrepancies were found compared to published drug guidelines, which could influence recommendations for therapy. Another limitation is that the analysis of interactions was done with only two drugs. It needs to be considered that multiple drug combinations could result in additional ADR effects. The sample size of this study was small, and most of the COVID-19 patients were asymptomatic. We excluded COVID-19 patients that were severely ill, as they were also treated with other drugs, and this would have affected our ADR analyses.

It still remains a challenge to design an effective, low-toxicity, and well-tolerated drug regimen for COVID-19 using off-label drugs [7, 13]. Our results have contributed to the modification of various aspects of the Cuban COVID-19 health guidelines. Additionally, our results have also provided data that could inform individualized therapies and could lead to better COVID-19 symptom management.

## 5. Conclusions

The prevalence of ADRs was high in Cuban COVID-19 patients that were enrolled in this study. The majority of the ADRs were drug-induced gastrointestinal disorders and general symptoms. Age, sex, and underlying diseases were the main independently associated factors for the occurrence of ADRs in patients. The reported characterization and incidence reflected the adverse reactions of patients with symptomatic and asymptomatic COVID-19, providing a reference for clinically safe medication.

## Figures and Tables

**Table 1:** Characteristics of patients with ADRs and without ADRs hospitalized at HEG-UCI, Pinar del Rio, and IPK, 2020.

Variables/groups		All patients	ADRs	Without ADRs	OR (95% CI)	*P*
*N*		200	110	90		
Age (years) Median (CI 95%)		46 (20–76)	56 (34–75)	41 (20–64)	2.36 (1.02–5.44)	**0.0059**
Patient sex	Male	106	46	60	0.40 (0.211–0.742)	**0.0038**
	Female	94	64	30		
Ethnicity	Caucasian	145	80	65	—	0.1110
	Mixed race	23	17	5		
	Black	32	13	20		
Body mass index median ± SD (kg/m^2^)		21.2 ± 2.3	20.5 ± 1.2	22.1 ± 2.0	—	0.062
Underlying diseases (chronic heart disease, asthma, stroke, hypertension, and diabetes)						
Yes		139	98	41	0.209 (0.11–0.40)	**0.0001**
No		61	12	49	—	
Asymptomatic COVID-19						
Yes		65	32	43	—	0.421
No		135	78	50	—	
Toxic habits (smoking status active alcoholism)						
Yes		75	58	44	—	0.08
No		125	52	46	—	

Source: completed surveys that were accessed in a digital database and printed surveys. ADR, adverse drug reaction. Data are *n* (%) or mean or standard deviation (SD). *P* values in bold italic show variables that are statistically significant. OR odd ratio. CI confidential interval. Underlying diseases included hypertension, cardiovascular disease, cerebrovascular disease, diabetes, and allergy. Source: completed surveys accessed in a digital database and printed surveys. HEG-UCI: Hospital Ernesto Guevara at University of Informatics' Science (HEG-UCI acronyms in Spanish) IPK-Institute Pedro Kourí. Period of hospitalization from April to July 2020.

**Table 2:** Adverse reactions by intensity and frequency.

Frequency	Intensity *N* (%)		Total *N* (%)
	Moderate	Serious	
Frequent	88 (83.9)	5 (3.6)	93 (87.6)
Rare	8 (5.8)	3 (1.8)	11 (8.0)
Occasional	4 (2.9)	2 (1.4)	6 (4.4)
No described	—	—	
Total *n* (%)	100 (92.7)	10 (7.3)	110 (100.0)

Source: completed surveys that were accessed in a digital database and printed surveys. *N* number of patients. % percent.

**Table 3:** Frequencies of DDI by drug, together with the description of each interaction according to: mechanism, risk level, effect, and recommendations.

Drugs	Risk level	*N* (%)	Interaction (potential)	Mechanism/effect	
Hydrochlorothiazide	C	16 (26.2)	Lopinavir + ritonavir Chloroquine Interferon alpha-2b	Hydrochlorothiazide can cause electrolyte disturbances and thereby increase the risk of QT interval prolongation. Thus, caution and electrolyte monitoring are needed when administering lopinavir and chloroquine The risk or severity of neutropenia and thrombocytopenia can be increased when hydrochlorothiazide is combined with interferon alpha-2b	
Antihypertensive					
Amlodipine	C	7 (11.47)	Lopinavir + ritonavir Chloroquine	Chloroquine will increase the level or effect of amlodipine by affecting hepatic/intestinal enzyme CYP3A4 metabolism. Avoid or use an alternate drug Lopinavir will increase the level or effect of amlodipine by affecting hepatic/intestinal enzyme CYP3A4 metabolism. Use caution/monitor	
Propranolol	C	1 (1.64)	Lopinavir + ritonavir	Propranolol is metabolized by 3 routes (aromatic hydroxylation by CYP2D6, N-dealkylation, followed by side chain hydroxylation via CYPs 1A2, 2C19, 2D6, and direct glucuronidation). Lopinavir could potentially increase propranolol concentrations, although to a moderate extent	
Antianginas-coronary vasodilator					
Dinitrate of isosorbide	D	1 (1.64)	Lopinavir + ritonavir	Lopinavir will increase the level or effect of isosorbide dinitrate by affecting hepatic/intestinal enzyme CYP3A4 metabolism. Avoid or use an alternate drug	
Anti-arrhythmic					
Verapamil	D	1 (1.64)	Lopinavir + ritonavir	Lopinavir will increase the level or effect of verapamil by affecting hepatic/intestinal enzyme CYP3A4 metabolism. Avoid or use an alternate drug	
Propafenone	C	1 (1.64)	Lopinavir + ritonavir Chloroquine	Propafenone is metabolized mainly by CYP2D6 and to a lesser extent by CYP1A2 and CYP3A4. Use with caution and therapeutic monitoring of propafenone is recommended. Note that both drugs (ritonavir-lopinavir) have risks of QT prolongation and/or TdP Chloroquine will increase the level or effect of propafenone by affecting hepatic enzyme CYP2D6 metabolism. Use caution/monitor. Chloroquine increases the toxicity of propafenone by QTc interval. Use caution/monitor	
Digoxin	C	1 (1.64)	Lopinavir + ritonavir	P-glycoprotein inhibition. Prolonged PR clinical and therapeutic drug monitoring of digoxin concentrations is recommended	
Platelet antiaggregate					
Clopidogrel	D	1 (1.64)	Lopinavir + ritonavir	Inhibition of CYP3A4, (2B6, 2C9, and 1A2) decreased the AUC and cmax of clopidogrel's active metabolite by 51% and 48%. Thrombotic risk, an alternative antiplatelet agent should be considered	
Hypoglycemic					
Glimepiride	C	2 (3.4)	Lopinavir + ritonavir Chloroquine		Glimepiride is mainly metabolized by CYP2C9: lopinavir decreases the effects of glimepiride as a modest inducer of CYP2C9. Risk of hyperglycemia due to insulin resistance with protease inhibitors as chloroquine may enhance the effects of a hypoglycemic treatment, a decrease in doses of insulin or other antidiabetic drugs may be required
Glibenclamide	C	1 (1.64)	Lopinavir + ritonavir Chloroquine		Glibenclamide is mainly metabolized by CYP3A4 and, to a lesser extent, by CYP2C9. Lopinavir/ritonavir could potentially increase glibenclamide concentrations by CYP3A4 and 2D6 inhibition. Risk of hypoglycaemia as chloroquine may enhance the effects of a hypoglycemic treatment, a decrease in doses of insulin or other antidiabetic drugs may be required
Antihistaminic					
Loratadine	C	5 (8.19)	Lopinavir + ritonavir Chloroquine	Lopinavir-ritonavir oral will increase the level or effect of loratadine oral by altering drug metabolism Monitoring is required chloroquine metabolism can be decreased when combined with loratadine	
Cimetidine	D	1 (1.64)	Lopinavir + ritonavir	Cimetidine increases levels of chloroquine by decreasing metabolism. Avoid or use an alternate drug Cimetidine will increase the level or effect of lopinavir by affecting hepatic/intestinal enzyme CYP3A4 metabolism. Avoid or use an alternate drug	
Corticosteroid					
Prednisone	C	7 (11.47)	Lopinavir + ritonavir	Prednisone will decrease the level or effect of lopinavir by affecting hepatic/intestinal enzyme CYP3A4 metabolism. Use caution/monitor	
Hydrocortisone	C	1 (1.64)	Lopinavir + ritonavir	Hydrocortisone will decrease the level or effect of lopinavir by affecting hepatic/intestinal enzyme CYP3A4 metabolism. Use caution/monitor	
Anticonvulsant					
Carbamazepine	D	2 (3.41)	Lopinavir Chloroquine	Carbamazepine will decrease the level or effect of lopinavir by affecting hepatic/intestinal enzyme CYP3A4 metabolism. Avoid or use an alternate drug Carbamazepine will decrease the level or effect of chloroquine by affecting hepatic/intestinal enzyme CYP3A4 metabolism. Use caution/monitor	
Lamotrigine	C	1 (1.64)	Lopinavir + ritonavir	Lopinavir will decrease the level or effect of lamotrigine by increasing metabolism. Use caution/monitor. Combination may decrease lamotrigine levels by 50%. Adjust lamotrigine dose as needed when starting, stopping, or changing lopinavir/ritonavir dose	
Sodium valproate	D	2 (3.41)	Lopinavir + ritonavir	Ritonavir may decrease the blood levels and effects of valproate. Moderately clinically significant. Usually avoid combinations; use it only under special circumstances	
Clonazepam	D	3 (4.92)	Lopinavir + ritonavir	Lopinavir increases levels of clonazepam by affecting hepatic/intestinal enzyme CYP3A4 metabolism. Modify therapy/monitor closely. Potential for increased toxicity. Use alternatives if available. Consider lowering benzodiazepine dose	
Hypnotic/sedatives					
Chlordiazepoxide	D	2 (3.41)	Lopinavir + ritonavir	Lopinavir increases levels of chlordiazepoxide by affecting hepatic/intestinal enzyme CYP3A4 metabolism. Modify therapy/monitor closely. Use alternatives if available. Consider lowering benzodiazepine dose	
Alprazolam	D	1 (1.64)	Lopinavir + ritonavir	Lopinavir will increase the level or effect of alprazolam by affecting hepatic/intestinal enzyme CYP3A4 metabolism. Avoid or use an alternate drug	
Diazepam	D	1 (1.64)	Lopinavir + ritonavir	Lopinavir increases levels of diazepam by affecting hepatic/intestinal enzyme CYP3A4 metabolism. Modify therapy/monitor closely. Use alternatives if available	
Clobazam	D	1 (1.64)	Lopinavir + ritonavir Chloroquine	CYP3A4 inhibition by lopinavir/ritonavir may increase clobazam exposure and prolong the duration of its effect, clobazam will increase the level or effect of chloroquine by affecting hepatic enzyme CYP2D6 metabolism. Use caution/monitor. Lower doses of drugs metabolized by CYP2D6 may be required when used concomitantly	
Antidepressant					
Amitriptyline	D	2 (3.41)	Lopinavir + ritonavir chloroquine	Lopinavir/ritonavir could potentially increase amitriptyline exposure although to a moderate extent. No a priori dosage adjustment is recommended but monitor adverse effects Chloroquine increases the toxicity of amitriptyline by QTc interval. Use caution/monitor	
Sertraline	C	1 (1.64)	Lopinavir + ritonavir	Lopinavir increases levels of sertraline by affecting hepatic/intestinal enzyme CYP3A4 metabolism. Use caution/monitor. Potential for increased toxicity. Chloroquine increases the toxicity of sertraline by QTc interval. Minor/significance unknown	
Antipsychotic					
Pimozide	X	1 (1.64)	Lopinavir + ritonavir Chloroquine	Chloroquine increases toxicity of pimozide by QTc interval. Contraindicated Lopinavir will increase the level or effect of pimozide by affecting hepatic/intestinal enzyme CYP3A4 metabolism. Contraindicated	
Nonsteroidal anti-inflammatory drugs (NSAIDs)					
Metamizole (dipirone)	C/D	9 (14.75)	Lopinavir + ritonavir	Metamizole is a moderate inducer of CYP3A4 and a strong inducer of CYP2B6. The risk or severity of nephrotoxicity can be increased when lopinavir is combined with metamizole also increased risk of haematological toxicity had to be considered	
Opioid					
Tramadol	C	1 (1.64)	Lopinavir + ritonavir Chloroquine	Lopinavir will increase the level or effect of tramadol by affecting hepatic/intestinal enzyme CYP3A4 metabolism Tramadol is metabolized by multiple CYPs 3A4, 2B6, and 2D6. Chloroquine is a moderate inhibitor of CYP2D6. It will also increase the level or effect of tramadol	
Antimicrobial					
Ciprofloxacin	C	1 (1.64)	Chloroquine	Coadministration of ciprofloxacin (500 mg) with chloroquine (600 mg) decreased ciprofloxacin cmax and AUC by 18% and 43%, respectively. Chloroquine and ciprofloxacin both increase QTc interval. Use caution/monitor	
Oral anticonceptive					
Levonogestrel	D	2 (3.27)	Lopinavir + ritonavir	Levonorgestrel is metabolized by CYP3A4 and is glucuronidated to a minor extent; coadministration is predicted to increase levonorgestrel	

Source: completed surveys that were accessed in a digital database and printed surveys. ADR, adverse drug reaction. Risk level: *X* (contraindicated association), *D* (consider modification of therapy), and C (monitoring therapy). CYP450, Cytochrome 450. AUC, area under curse. *C*_max_: drug maximum (or peak) serum concentration that achieves in a specified compartment. QTc: the interval is a measurement made on an electrocardiogram in relation to cardiac frequency using the beginning of the QRS complex to the end of the *T* wave. TdP: torsades-de pointes (French for “twisting of the points”) is one of several types of life-threatening heart rhythm disturbances. mg: miligram.

## Data Availability

There are specific legal and ethical requirements related to Cuban populations that prohibit public sharing of a dataset. Data contains personal information on human subjects. Confidentiality and any specific access restrictions are typically specified by an Institutional Review Board.

## References

[1] Wang R., Xu Q., Li L., Wang X., Jiang S., Lu X. (2020). Pharmacological care strategy for antivirals in patients with COVID-19 complicated by underlying disorders. *Chinese Journal of Hospital Pharmacy*.

[2] Zhou P., Yang X. L., Wang X. G. (2020). A pneumonia outbreak associated with a new coronavirus of probable bat origin. *Nature*.

[3] WHO (2020). Coronavirus disease (Covid-2019).

[4] Dosch S. F., Mahajan S. D., Collins A. R. (2009). SARS coronavirus spike protein-induced innate immune response occurs via activation of the NF-*κ*B pathway in human monocyte macrophages in vitro. *Virus Research*.

[5] Muralidharan S., Mandrekar P. (2013). Cellular stress response and innate immune signaling: integrating pathways in host defense and inflammation. *Journal of Leukocyte Biology*.

[6] Azkur A. K., Akdis M., Azkur D. (2020). Immune response to SARS-CoV-2 and mechanisms of immunopathological changes in COVID-19. *Allergy*.

[7] Liu C., Zhou Q., Li Y. (2020). Research and development on therapeutic agents and vaccines for COVID-19 and related human coronavirus diseases. *ACS Central Science*.

[8] Guo Y. R., Cao Q. D., Hong Z. S. (2020). The origin, transmission and clinical therapies on coronavirus disease 2019 (COVID-19) outbreak - an update on the status. *Military Medical Research*.

[9] Jing Y., Diao L., Han L. (2021). Adverse events associated with potential drugs for COVID-19: a case study from real-world data. *Briefings in Bioinformatics*.

[10] Shuter J., Shuter J. (2008). Lopinavir/ritonavir in the treatment of HIV-1 infection: a review. *Therapeutics and Clinical Risk Management*.

[11] Cruz Barrios M. A., Rodríguez Montiel B. N., Furones Mourelle J. A., Alfonso Orta I., Rodríguez Piñero D. (2012). Eventos adversos observados después del tratamiento con factor de transferencia. *Revista Cubana de Salud Pública*.

[12] autores C. D. (2020). *Protocolo de actuación nacional para el enfrentamiento a la COVID 19, Versión 1.4*.

[13] Desai M. K. (2020). Pharmacovigilance and assessment of drug safety reports during COVID 19. *Perspectives in clinical research*.

[14] Bucşa C., Farcaş A., Cazacu I. (2013). How many potential drug-drug interactions cause adverse drug reactions in hospitalized patients?. *European Journal of Internal Medicine*.

[15] Parasa S., Desai M., Thoguluva Chandrasekar V. (2020). Prevalence of gastrointestinal symptoms and fecal viral shedding in patients with coronavirus disease 2019: a systematic review and meta-analysis. *JAMA Network Open*.

[16] de autores C. (2018). *Cuadro básico de medicamentos y productos naturales*.

[17] Cao B., Wang Y., Wen D. (2020). A trial of lopinavir-ritonavir in adults hospitalized with severe covid-19. *New England Journal of Medicine*.

[18] Geer M. I., Koul P. A., Tanki S. A., Shah M. Y. (2016). Frequency, types, severity, preventability and costs of Adverse Drug Reactions at a tertiary care hospital. *Journal of Pharmacological and Toxicological Methods*.

[19] Olsson S., Pal S. N., Dodoo A. (2015). Pharmacovigilance in resource-limited countries. *Expert Review of Clinical Pharmacology*.

[20] Tarragó Portelles S. S., Gravier Hernández R., Gil del Valle L. (2018). La farmacovigilancia en Cuba y la infranotificaciones de reacciones adversas a los medicamentos. *Horizonte sanitario*.

[21] Narumol J., Arunrot P., Krska J. (2015). Survey of patients’ experiences and their certainty of suspected adverse drug reactions. *International Journal of Clinical Pharmacy*.

[22] Arrivillaga M., Correa D., Salazar I. C. (2003). Creencias sobre la salud y su relación con las prácticas de riesgo o de protección en jóvenes universitarios. *Colombia Médica*.

[23] WHO (2020). The use of the who-umc system for standardised case causality assessment. https://www.who.int/medicines/areas/quality_safety/safety_efficacy/WHOcausality_assessment.pdf.

[24] Nebeker J. R., Barach P., Samore M. H. (2004). Clarifying adverse drug events: a clinician’s guide to terminology, documentation, and reporting. *Annals of Internal Medicine*.

[25] Drugs M. (2020). Drug Interactions Checker. https://www.drugs.com/interaction/list/.

[26] Liverpool U. D. (2020). Herramienta online de interacciones medicamentosas de la Universidad de Liverpool. https://www.covid19-druginteractions.org/.

[27] Sun J., Deng X., Chen X. (2020). Incidence of adverse drug reactions in COVID-19 patients in China: an active monitoring study by hospital pharmacovigilance system. *Clinical Pharmacology & Therapeutics*.

[28] Zekarias A., Watson S., Vidlin S. H., Grundmark B. (2020). Sex differences in reported adverse drug reactions to COVID-19 drugs in a global database of individual case safety reports. *Drug Safety*.

[29] Wang J. T., Sheng W. H., Fang C. T. (2004). Clinical manifestations, laboratory findings, and treatment outcomes of SARS patients. *Emerging Infectious Diseases*.

[30] Melo J. R. R., Duarte E. C., Moraes M. V. d., Fleck K., Silva A. S. d. N. E., Arrais P. S. D. (2021). Adverse drug reactions in patients with COVID-19 in Brazil: analysis of spontaneous notifications of the Brazilian pharmacovigilance system. *Cadernos de Saúde Pública*.

[31] Maza-Larrea J., Rosado-Hernández F., Rojas-Velasco G. (2021). Evaluación del perfil de seguridad del medicamento lopinavir/ritonavir (Lpv/r) en los pacientes sospechosos o confirmados por COVID-19. *Rev. OFIL•Ilaphar*.

[32] Gevers S., Kwa M. S. G., Wijnans E., van Nieuwkoop C. (2020). Safety considerations for chloroquine and hydroxychloroquine in the treatment of COVID-19. *Clinical Microbiology and Infections*.

[33] Sierra B., Triska P., Soares P. (2017). OSBPL10, RXRA and lipid metabolism confer African-ancestry protection against dengue haemorrhagic fever in admixed Cubans. *PLoS Pathogens*.

[34] Kojima T., Matsui T., Suzuki Y. (2020). Risk factors for adverse drug reactions in older inpatients of geriatric wards at admission: multicenter study. *Geriatrics and Gerontology International*.

[35] Brandariz-Nuñez D., Correas-Sanahuja M., Guarc E., Picón R., García B., Gil R. (2020). Potential drug-drug interactions in COVID 19 patients in treatment with lopinavir/ritonavir. *Medicina Clínica*.

[36] Alomar M. J. (2014). Factors affecting the development of adverse drug reactions (Review article). *Saudi Pharmaceutical Journal*.

[37] Rehman S., Ravinayagam V., Nahvi I. (2021). Immunity, sex hormones, and environmental factors as determinants of COVID-19 disparity in women. *Frontiers in Immunology*.

